# Alzheimer’s Disease-like Pathological Features in the Dorsal Hippocampus of Wild-Type Rats Subjected to Methionine-Diet-Evoked Mild Hyperhomocysteinaemia

**DOI:** 10.3390/cells12162087

**Published:** 2023-08-17

**Authors:** Maria Kovalska, Petra Hnilicova, Dagmar Kalenska, Marian Adamkov, Libusa Kovalska, Jan Lehotsky

**Affiliations:** 1 Department of Histology and Embryology, Jessenius Faculty of Medicine, Comenius University in Bratislava, 03601 Martin, Slovakia; maria.kovalska@uniba.sk (M.K.); marian.adamkov@uniba.sk (M.A.); 2 Biomedical Center Martin, Jessenius Faculty of Medicine, Comenius University in Bratislava, 03601 Martin, Slovakia; petra.hnilicova@uniba.sk; 3 Department of Anatomy, Jessenius Faculty of Medicine, Comenius University in Bratislava, 03601 Martin, Slovakia; dagmar.kalenska@uniba.sk; 4 Clinic of Stomatology and Maxillofacial Surgery, Jessenius Faculty of Medicine, Comenius University in Bratislava, 03601 Martin, Slovakia; libusa.kovalska@uniba.sk; 5 Department of Medical Biochemistry, Jessenius Faculty of Medicine, Comenius University in Bratislava, 03601 Martin, Slovakia

**Keywords:** rat, dorsal hippocampus, neurodegeneration, Alzheimer’s disease-like pathology, methionine diet, ^1^H MRS

## Abstract

Multifactorial interactions, including nutritional state, likely participate in neurodegeneration’s pathogenesis and evolution. Dysregulation in methionine (Met) metabolism could lead to the development of hyperhomocysteinaemia (hHcy), playing an important role in neuronal dysfunction, which could potentially lead to the development of Alzheimer’s disease (AD)-like pathological features. This study combines proton magnetic resonance spectroscopy (^1^H MRS) with immunohistochemical analysis to examine changes in the metabolic ratio and histomorphological alterations in the dorsal rat hippocampus (dentate gyrus—DG) subjected to a high Met diet. Male Wistar rats (420–480 g) underwent hHcy evoked by a Met-enriched diet (2 g/kg of weight/day) lasting four weeks. Changes in the metabolic ratio profile and significant histomorphological alterations have been found in the DG of hHcy rats. We have detected increased morphologically changed neurons and glial cells with increased neurogenic markers and apolipoprotein E positivity parallel with a diminished immunosignal for the N-Methyl-D-Aspartate receptor 1 in hHcy animals. A Met diet induced hHcy, likely via direct Hcy neurotoxicity, an interference with one carbon unit metabolism, and/or epigenetic regulation. These conditions lead to the progression of neurodegeneration and the promotion of AD-like pathological features in the less vulnerable hippocampal DG, which presents a plausible therapeutic target.

## 1. Introduction

Neurodegenerative diseases represent a significant portion of the neurological public health encumbrance worldwide [1,2,3,4,5,6].

Currently, due to the limited effectiveness of Alzheimer’s disease (AD) treatment, alternative therapeutic options are being continuously examined [5,7]. With the development of nutritional epigenetics, nutraceuticals, and dietary interventions [5,6,8], it has been established that the disruption of methionine (Met) metabolism significantly contributes to the development and progression of neurodegenerative diseases [2,9]. Met is an essential amino acid containing sulphur and must be acquired through external dietary sources [3,4,5,6]. The consumption of a diet high in Met has been observed to influence brain function and initiate neurotoxic effects, leading to neurodegeneration akin to dementia [6]. Excessive or insufficient Met intake can adversely affect other organ systems [10,11,12], including the brain [6]. Diets that are high in Met, low in folate, and deficient in vitamin B6/B12 have been associated with various issues such as vascular leakage, cerebral vascular dysfunction, short-term memory loss, and neurodegeneration [13,14].

Moreover, as experimentally proven, high protein intake, which is abundant in Met, or disruptions in Met metabolism lead to an increase in a toxic sulphur intermediate called homocysteine (Hcy) in the circulating plasma [10,11,15]. Hcy is produced as a metabolic byproduct during the conversion of Met to cysteine. Elevated levels of Hcy in the plasma, known as hyperhomocysteinaemia (hHcy), represent well-established risk factors for cardiovascular and cerebrovascular disorders [10,11,13]. Studies on various tissues have confirmed the clinical significance of elevated total Hcy levels in the plasma [10,11,15]. Several studies have shown an association of hHcy with increased neuroinflammation and significant cognitive deficits [11,12,13,14,15,16,17,18,19]. Remarkably, the development of progressive atherosclerosis, acute ischemic stroke, and neurodegenerative features, such as dementia, was also shown to be associated with hHcy [13,17,18,19]. However, the exact toxic mechanism affecting neuronal tissue is still not fully unravelled.

There is only little information on how, and to which extent, a Met-rich diet can initiate and progress neurodegeneration in selective vulnerable or more resistant brain areas. The hippocampus is particularly susceptible to neurodegenerative processes [20], affecting the distinct forms of plasticity, learning, and memory [21]. Our prior studies using the rat hHcy model evoked by a Met-enriched diet [22,23] showed significant cerebral dysregulations in the hippocampus’s cornu Ammonis 1 (CA1) region. These dysregulations were evident at the level of cerebral metabolites, which were associated with accelerated neurodegeneration and a decline in the animal’s behavioural profile, as confirmed through histological analysis. Additionally, our prior findings indicated that the combination of hHcy with subsequent ischemia–reperfusion insult exacerbates neurodegenerative processes, resulting in the development of Alzheimer’s disease (AD)-like pathology [24].

In the context of the typical amnestic dementia syndrome, the dentate gyrus (DG), especially its granule cells, shows resilience to the formation of AD pathology [25]. The DG plays a crucial role in the trisynaptic pathway, serving as a major station that receives input from the cortex and relays it to the hippocampus. Notably, DG granule cells excite neurons in cornu Ammonis 3 (CA3), which further project to CA1 neurons [18,26,27,28]. Remarkably, the DG is one of the two primary neurogenic regions in the postnatal human brain and has been extensively investigated in the context of neurogenesis [21]. On the other hand, significant pathologies occur in the DG as a part of the complex neurodegeneration process [27,28]. Furthermore, as previously demonstrated, hHcy exhibits neurotoxic properties by functioning as a potent agonist for N-Methyl-D-Aspartate receptors (NMDARs), which are prominently expressed in the DG of the hippocampus [29].

Using novel methodological approaches, we focused in this work on whether a Met-enriched diet (inducing hHcy) can also affect a less vulnerable hippocampal DG area in rats. In vivo magnetic resonance (MR) spectroscopy was used to explore tissue metabolic profiles. Histomorphological and neurogenic pattern alterations were observed in the rat hippocampal DG following the impact of hHcy induced by a high Met diet, indicating the development of AD-like pathology.

## 2. Materials and Methods

### 2.1. Experimental Procedures with Mild hHcy Induction

The animals in this study were handled in compliance with the guidelines for Animal Care and Health of the State Veterinary and Food Department of Slovakia (approval number 3033-3/2020-220 for animal experiments). The study was conducted by Directive 2010/63/EU of the European Parliament and the Council on the Protection of Animals Used for Scientific Purposes.

**Figure 1:**
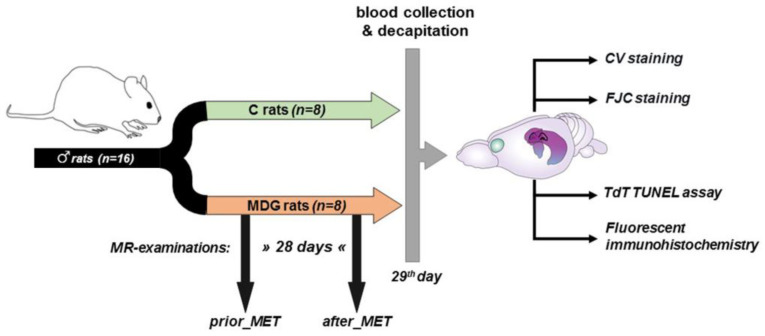
Scheme of experimental procedures (partially created with https://app.biorender.com/ (accessed on 26 June 2023).

Because of the antioxidant [30], anti-inflammatory [31], and neuroprotective effects [32,33,34] of oestrogen, we employed adult male Wistar rats (Dobra Voda, Bratislava, Slovakia) for this study. The rats were aged 4–6 months and weighed 420–480 g (with a mean body weight of 450 g). The total number of rats in the study was 16. They were housed in air-conditioned rooms under standard conditions, with a temperature of 22 ± 2 °C and a 12 h day/night cycle. Food and water were available freely and without restrictions. The experimental procedures were carried out following the scheme depicted in Figure 1.

Eight of the sixteen rats were subjected to a Met-enriched diet (MDG rats) for 28 days and underwent MR examination both before and after the Met diet (prior_MET, after_MET). Meanwhile, the control group (C rats, *n* = 8) did not receive the Met diet and was not exposed to MR procedures. We maintained the same conditions and mild hHcy induction throughout the experiment, consistent with our prior papers [22,23]. On day 0, we measured each animal’s drink water volume individually (each animal spent 24 h in an individual box). Both experimental groups consumed an average daily volume of 45.11 ± 4.27 mL. Subsequently, we housed two animals per cage (in accordance with EU directive 2010/63/EU) until the end of the experiment. After assessing the daily water intake, rats underwent mild hHcy induction as described in our previous studies [22,23,24]. The Met (L-methionine, Sigma-Aldrich, Darmstadt, Germany) was provided in drinking water at a dose of 2 g/kg of animal weight/day, following Xu et al.’s protocol [17]. The daily volume of Met-enriched water consumed was monitored and measured. The animals’ weights were recorded on days 0, 3, 7, 14, 21, and 28 of the experiment. On the 29th day, after the last Met administration but not later than 10 h, blood samples (1.0–1.5 mL) were obtained from the retroorbital venous plexus. After collecting the blood samples, they were promptly cooled on ice, centrifuged, and the resulting supernatant—plasma—was stored at −80 °C. We assessed the total plasma Hcy levels, following established protocols from our previous studies [22,23,24] as well as other experimental [14,15,17] and clinical works [35,36] for evaluating the mild hHcy induced by the Met dietary treatment.

On day 29, both experimental groups were decapitated, and their brains were carefully dissected from the skulls and prepared for future analysis (Figure 1).

### 2.2. MR Examination in Living Subjects

We conducted in vivo MR examination using a 7 T Bruker BioSpec small animal MR scanner (Bruker BioSpin MRI, Ettlingen, Germany) equipped with a 4-element surface array coil. Throughout the scanning, animals were anaesthetised (2–4% sevoflurane in medical O2), heated via a water-heated bed, and controlled regarding their body temperature and respiratory rates.

**Figure 2:**
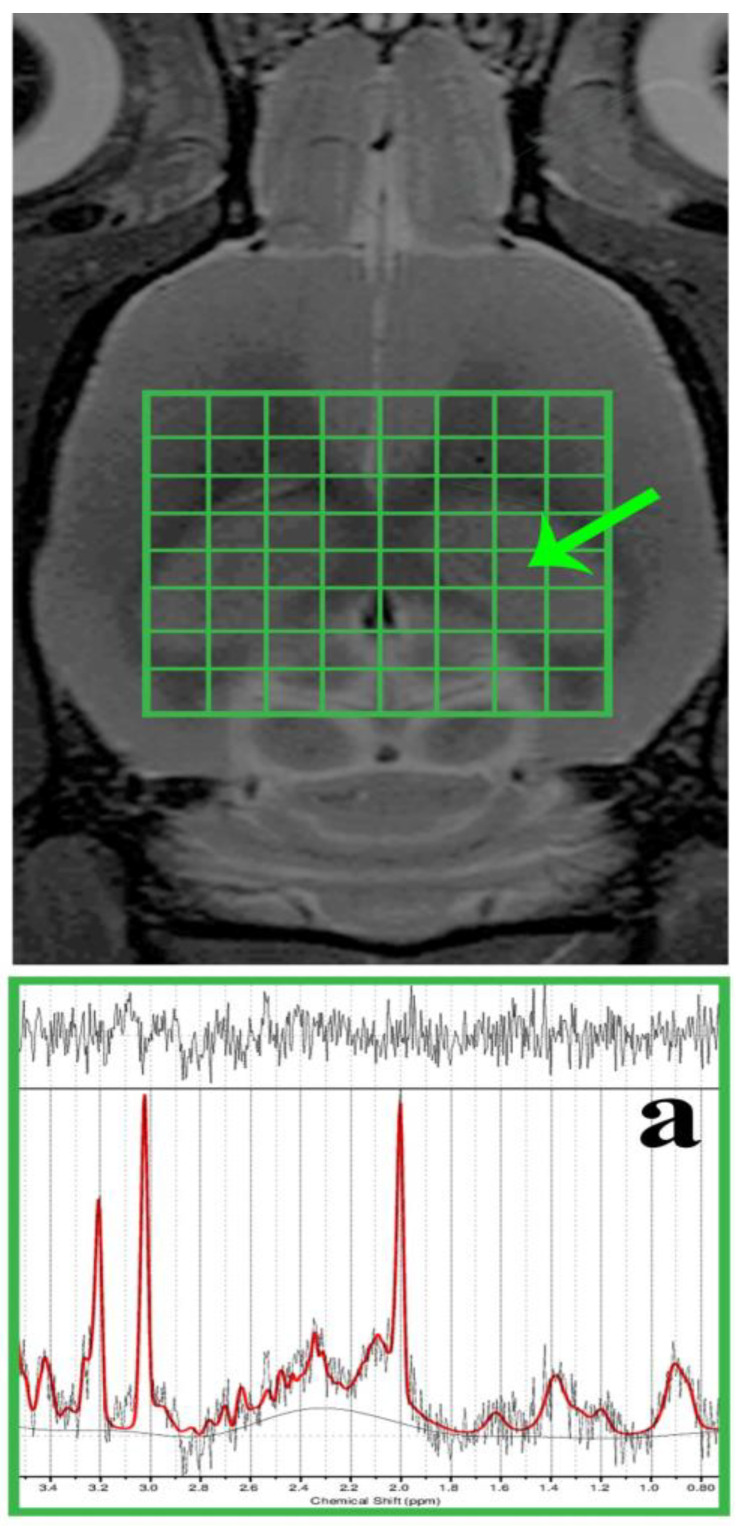
In vivo, ^1^H MRS was conducted in the rat brain’s dorsal hippocampus. The morphological T2-weighted MR images display the location of the CSI grid (indicated in green) with the size of the voxel as follows: 2.75 × 2.75 × 2 mm^3^, encompassing the dorsal hippocampus (outlined by the peripheral green line/a). Additionally, representative in vivo ^1^H MRS spectra from the rat’s dorsal hippocampus (a) were evaluated using LCModel.

MR spectroscopy (MRS) voxels (including the shimming volumes) were positioned in the required brain regions but outside B0 distortions. The dorsal hippocampal area (DG) was subjected to spectroscopic data acquisition using the two-dimensional chemical shift imaging (CSI) method (Figure 2) with a PRESS (point-resolved spectroscopy) pulse sequence. The acquisition parameters were as follows: 8 × 8 voxel matrix; 2.75 × 2.75 × 2 mm^3^ real voxel size; 22 × 22 mm^2^ FOV; TR/TE = 1500/20 ms; 36 averages; 6 kHz acquisition bandwidth; 16 min measurement time. Measures were taken to compensate for eddy currents and B0 drift, along with the implementation of OVS (outer volume suppression) and VAPOR (variable pulse power and optimised relaxation delays) suppression. Using the cuboid shim volume, automatic adjustments were made to linear and second-order shims. The water peak’s average linewidth was measured at 14.8 ± 1.9 Hz.

During the spectroscopic data analysis, each DG voxel (Figure 2a) was evaluated using the LCModel software (version 6.3-1K; S. Provencher, Oakville, ON, Canada). All spectra achieved a signal-to-noise ratio (SNR) greater than 5 and a full width at half maximum (FWHM) less than 16 Hz. Afterwards, the concentrations of total N-acetyl aspartate (tNAA), myo-Inositol (mIns), total choline (tCho), and total creatine (tCr) were expressed in the following ratios: tNAA/tCr, mIns/tNAA, mIns/tCr, tCho/tNAA, and tCho/tCr.

### 2.3. Cresyl Violet (CV) Staining

Following Met diet treatment and MR analysis, on day 29, the control and MDG groups (*n* = 8 per group) were anaesthetised in an anaesthetic box through the spontaneous inhalation of 3.5% sevoflurane mixed with oxygen and nitrous oxide (33/66%). Sequentially, the animals were transcardially perfused with 0.1 mol/L phosphate-buffered saline (PBS, pH 7.4). After PBS perfusion, a transcardial rinse with 250–300 mL of 4% paraformaldehyde (PFA) in 0.1 mol/L PBS (pH 7.4) followed [23].

**Figure 3:**
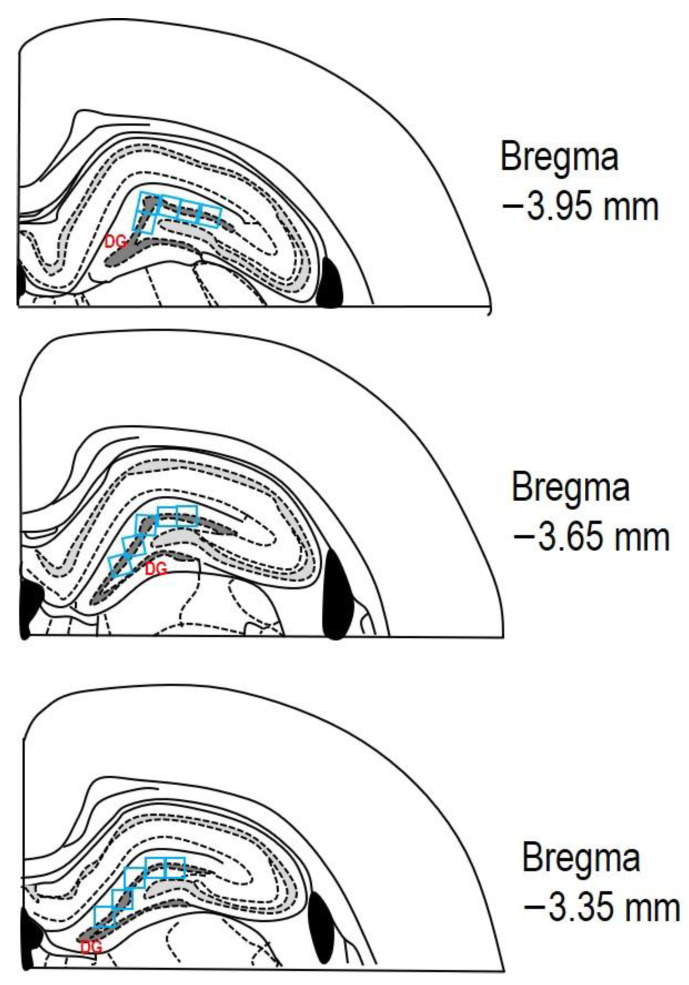
The schematic representation depicting three rat brain coronal sections (adapted from Paxinos and Watson [37]). The area of interest in the DG, where cell counting was performed, is marked by the dashed dark grey line. A counting grid of 0.3 × 0.3 mm (indicated by blue squares) was used within this region. Each section was analysed in the DG across at least three microscopic fields (blue squares). The stereotaxic coordinates from Bregma for the examined sections ranged from −3.35 mm to −3.95 mm.

After the perfusion of both solutions (PBS and PFA), all animals were decapitated, and their brains were taken out from the skull and immersed for 24 h in 4% PFA at 4 °C. Subsequently, the rat brains were transferred to 30% sucrose for the next 24–48 h at 4 °C. The brains were embedded in a cryoprotective medium (Killik, Bio Optica, Milano, Italy) and immediately frozen using a cryobar Shannon Cryotome E (Thermo Scientific, Waltham, MA, USA). The brains were then sectioned into 30 μm thick coronal slices. Stereotaxic coordinates of the rat brain were referenced from the atlas of Paxinos and Watson [37], with coordinates from Bregma approximately ranging from −3.35 mm to −3.95 mm. The sections were mounted on Superfrost Plus glass slides (Thermo Scientific, Waltham, MA, USA), dehydrated with descending grades of ethanol (100–70%; *v*/*v*), and washed with distilled water. The sections underwent staining with 0.1% (*w*/*v*) CV and were observed using a light microscope (AxioScope A1; Zeiss, Oberkochen, Germany). The degenerating neurons in the hippocampus’s DG were quantified by measuring cell density per field (see Figure 3 for reference).

### 2.4. Fluoro-Jade C (FJC) Staining

To mark neurodegeneration, FJC was utilised. Brain sections mounted on glass slides were heated to 50 °C for 30 min before the staining process. Subsequently, the slides were immersed in absolute alcohol (3 min), 70% alcohol (1 min), followed by a rinse in distilled water for an additional minute. Next, the slides were placed in a solution of 0.06% potassium permanganate (15 min), followed by a rinse in distilled water (2 min). After 2 h in the staining solution, the slides were washed thrice for 1 min each with distilled water. Finally, the slides were allowed to dry at room temperature and were coverslipped using Fluoro-mount™ Aqueous Mounting Medium (Sigma-Aldrich, Darmstadt, Germany) following standard protocols.

### 2.5. Terminal Deoxynucleotidyl Transferase (TdT) dUTP Nick-End Labelling (TUNEL) Assay

The in situ, nick-end labelling of nuclear DNA fragmentation in the sections was conducted according to the manufacturer’s instructions using a TUNEL detection kit (In Situ Cell Death Detection Kit, Fluorescein, Roche, Indianapolis, IN, USA). The labelling was performed in a dump chamber for 1 h in the dark at 37 °C. Prior to staining, positive control slides underwent treatment with 1 U μL–1 DNase I for 10 min at 37 °C in each experiment. Negative control slides were labelled parallel, but the enzyme TdT was omitted. The staining reaction (TUNEL) was halted with 2 × SSC (300 mmol/L NaCl, 30 mmol/L sodium citrate), and was followed by washing the slides with PBS (pH 7.2). Finally, the slides were mounted with Fluoromount™ Aqueous Mounting Medium (F4680, Sigma-Aldrich, Darmstadt, Germany). Afterwards, they were stored in the dark at 4 °C until a microscopic observation was conducted.

### 2.6. Fluorescent Immunohistochemistry

The primary antibodies employed in this study were as follows: NeuN (neural nuclei), NMDAR1 (N-methyl-D-aspartate receptor 1), DCX (doublecortin), bTub (III β-tubulin), Iba1 (Ionized calcium-binding adapter molecule 1), GFAP (glial fibrillary acidic protein), Nestin, and ApoE (apolipoprotein E). The Iba1/AIF1 antibody (1:100; mAb #17198; Cell Signaling Technology, Beverly, MA, USA) detected microglia. The anti-GFAP primary antibody (1:200; AB5804; Millipore, Burlington, MA, USA) was used as a specific astrocyte marker. The mature neuron marker NeuN (1:100; 24307S; Cell Signaling Technology, Beverly, MA, USA) rabbit antibody was also applied. As neurogenesis markers we used (i) an antibody against neural precursors—anti-Nestin (1:200; MA1-110; Santa Cruz Technology, Dallas, TX, USA), and (ii) an antibody against neuroblasts—anti-Doublecortin (DCX; 1:100; 4604; ThermoFisher Scientific, Waltham, MA, USA). The last two antibodies represent markers for early neurodegeneration associated with AD pathology. The specific glutamate receptors were stained using an anti-NMDAR1 antibody (1:100; ab134308; Abcam, Cambridge, UK). The last primary antibody was ApoE (1:100; 710265; CiteAb, Bath, UK). To examine the morphological features of the neuronal cytoskeleton, bTub (1:50; sc-5274; Santa Cruz Technology, Dallas, TX, USA) was also used.

Brain sections acquired from both groups (C and MDG were prepared following the previously described procedure) were permeabilised using 0.1% Triton X-100 and pre-blocked with 10% BSA (60 min). Subsequently, overnight incubation at 4 °C was performed on the brain sections with a solution of primary antibodies, which were appropriately diluted in 0.1% Triton X-100 supplemented with 10% BSA.

The fluorescent detection was accomplished using Alexa Fluor 488 goat-anti-mouse IgG (A11001, 1:100, Life Technologies, Beverly, MA, USA)-conjugated secondary antibody, and Alexa Fluor 594 goat-anti-rabbit IgG (A11012, 1:100, Life Technologies, Beverly, MA, USA)-conjugated secondary antibody. The brain sections were mounted using Fluoromount-G^®^ medium, which contained DAPI (4′, 6-diamidino-2-phenylindole; CA 0100-20, SouthernBiotech, Carlsbad, CA, USA), following the manufacturer’s protocol. The absence of the primary antibody resulted in no immunoreactivity. The slides were examined using a light microscope (AxioScope A1; Zeiss, Oberkochen, Germany) and a confocal laser scanning microscope, Olympus FluoView FV10i (Olympus, Tokyo, Japan), specifically in the hippocampal DG area. For light microscopy, objectives of 10× and 40× magnification were utilised. For fluorescent microscopy, an objective of 10× with zoom up to 400× magnification was used, equipped with filters for FITC (fluorescein isothiocyanate) for the observation of FJC and TUNEL methods (excitation: 495 nm and emission: 519 nm), Alexa Fluor 488 for examination GFAP, Nestin, NMDAR1 and ApoE (excitation: 499 nm and emission: 520 nm), and Alexa Fluor 594 for the detection of Iba1, DCX, NeuN, and bTub (excitation: 590 nm and emission: 618 nm). Image capture from the light microscope was accomplished with ZEN 2 core imaging software (Zeiss, Oberkochen, Germany). The confocal microscope utilised the following software: Olympus Fluoview FV10-ASW software, version 02.01 (Olympus, Tokyo, Japan), Quick Photo Micro software, version 2.3 (Promicra, Prague, Czech Republic), and the images were processed using Adobe Photoshop CS3 Extended, version 10.0 for Windows (Adobe Systems, San Jose, CA, USA).

### 2.7. Quantitative Image Analysis

Serial coronal brain sections (30 μm thick) were collected, with every 2nd section included (totalling 600 μm in the range between −3.35 mm and −3.95 mm from Bregma), and these sections were subsequently processed for staining.

For quantitative image analyses, two skilled observers, working independently and unaware of the experimental conditions, counted the cells in the hippocampal DG region, as indicated in Figure 3. The counting was performed on three random microscopic fields in a double-blind manner.

Subsequently, the results from 6 sections per animal were combined and then multiplied by 3 (representing three different microscopic fields of each DG area) to determine the total count of degenerative or positive cells in the subgranular and granular layers, as well as the hilus.

The contrast and brightness of each image file were standardised using Adobe Photoshop CS3 Extended, version 10.0 for Windows (Adobe Systems, San Jose, CA, USA). The coronal brain section images from both experimental groups (C and MDG) were exported in a tiff format and analysed using ImageJ software (NIH, Bethesda, MD, USA). The RGB channels were converted to 8-bit grayscale images, and threshold levels were modified within the range of 13 to 255 pixels. A particle analysis was performed with size restrictions ranging from 0 cm^2^ to infinity, without specifying morphology. For histomorphological analysis in three different areas of the DG region in the rat hippocampus, the following counting parameters were used: sampling grid size of 1 × 3 mm, and counting frame size of 0.3 × 0.3 mm. Cell counting was conducted manually with the assistance of automatic counting correlations, which accounted for a 3–5% variation in the results. The cell counts are quantified as the total number of cells per optical field (0.3 × 0.3 mm).

### 2.8. Statistical Analysis

A statistical analysis for spectroscopic data was conducted using the SPSS software package (Version 15.0; Chicago, IL, USA). The data distribution was assessed for normality using the Kolmogorov–Smirnov normality test. To analyse metabolic differences in the dorsal hippocampus resulting from the Met diet (i.e., prior_MET vs. after_MET), a 2-tailed paired *t*-test was performed, with a significance level set at *p* < 0.05.

GraphPad Prism software, version 6.01 for Windows (La Jolla, CA, USA), was used for data obtained from brain section image analysis. The normality of data distribution was assessed using the Kolmogorov–Smirnov test. To evaluate the significance of mean group differences, an unpaired *t*-test with Welch correction was employed to compare the control (C) and MDG groups, with a significance level set at *p* < 0.05.

## 3. Results

### 3.1. MR Spectroscopic Analysis

In the dorsal hippocampus, ^1^H MRS was utilised to quantify significant metabolites. The analysis revealed a reduction in both the tNAA/tCr and tCho/tCr ratios, while an increase was observed in the mIns/tNAA, mIns/tCr, and tCho/tNAA ratios between the prior_MET and after_MET groups. However, when examined for statistical significance, differences in the metabolic ratio between the prior_MET and after_MET did not show statistical significance (Table 1). Moreover, statistically significant changes were not detected between experimental groups for both tCho ratios (e.g., to tNAA and tCr).

### 3.2. Histomorphological Analysis

#### 3.2.1. CV, FJC, and TUNEL Analyses

**Figure 4:**
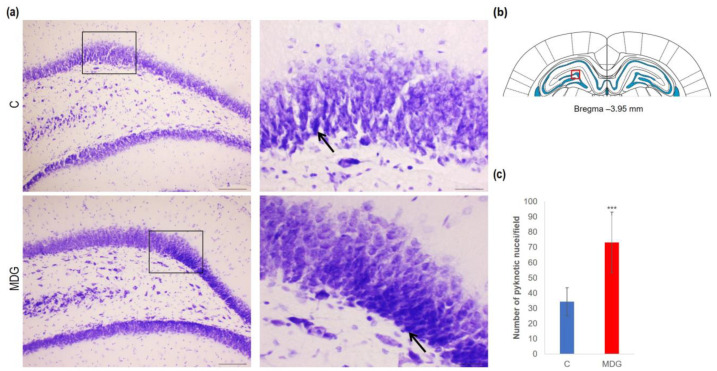
Illustrative micrographs of CV-stained brain sections were used for statistical analysis to evaluate changes in the number of disintegrated neurons in the DG area of the rat hippocampus. (**a**) DG region of the hippocampus displays the C and MDG groups at 10× magnification in the left row, with the corresponding groups at high (400×) magnification (rectangle) in the right row. Morphologically altered neurons with pyknotic nuclei are indicated by an arrow (*n* = 8 per group). The coronal rat brain section depicted in (**b**) has been redrawn from Paxinos and Watson [37], illustrating the DG photographed area (red rectangle). (**c**) The number of neurons displaying degeneration marks in the hippocampus’s DG region for the control and MDG groups. The significance of differences in the group means was assessed using an unpaired *t*-test. The results are expressed as the mean ± SD for each group, with a sample size of *n* = 8, *** *p* < 0.001 compared to the control group.

In order to explore the effect of hHcy on the count of disintegrated neurons as a potential indicator of neurodegeneration, we conducted CV, FJC, and TUNEL analyses on tissue slices from the DG region of the rat hippocampus. Using CV staining, we detected neurons with pyknotic nuclei, mainly in the granular layer of DG in the MDG animals. The number of cells with such morphological patterns reached a 2.14-fold increase in MDG compared to the controls (*p* < 0.001; Figure 3). Additionally, morphologically changed neurons (arrows) were shrunk, and tightly packed with pyknotic nuclei in the granular layer, likely due to neuronal tissue remodelling that can be identified in mild hHcy conditions (Figure 4).

**Figure 5:**
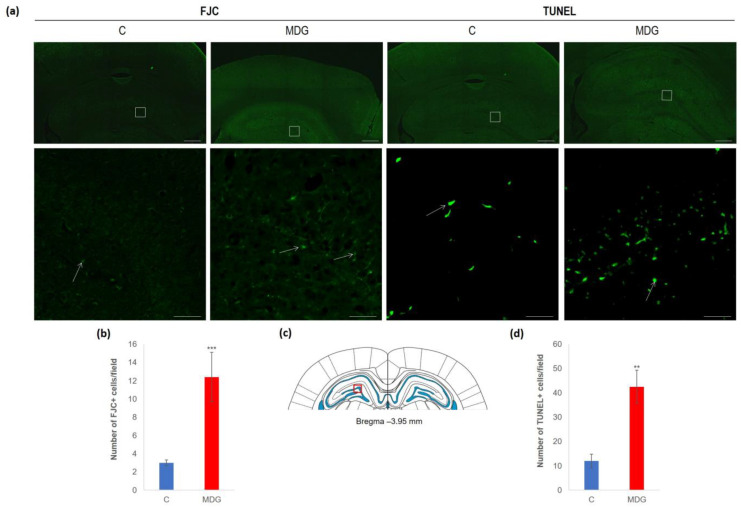
Illustrative micrographs of FJC and TUNEL staining in rat brain sections from the DG area of the hippocampus. (**a**) Fluorescent microphotographs of the hippocampus show the control and MDG groups at low magnification in the first line, and the second line signifies the corresponding groups at high magnification (rectangles). Arrows indicate FJC/TUNEL positivity. Scale bars = 500 and 50 μm; *n* = 8 per group. (**b**) The number of FJC+ neurons in the DG region of the hippocampus for the control and MDG groups. The FJC+ significance of differences in group means was assessed using an unpaired *t*-test. The results are expressed as the mean ± SD for each group, with a sample size of *n* = 8, *** *p* < 0.001 compared to the control group. The coronal rat brain section depicted in (**c**) has been redrawn from Paxinos and Watson [37], illustrating the DG photographed area (red rectangle). (**d**) The number of TUNEL+ neurons in the DG region of the hippocampus for the control and MDG groups. The TUNEL+ significance of differences in group means was assessed using an unpaired *t*-test. The results are expressed as the mean ± SD for each group, with a sample size of *n* = 8, ** *p* < 0.01 compared to the control group.

The impact of hHcy has also been detected with FJC staining, apparently used to detect neurodegeneration features. FJC staining shows remarkable differences between the controls and the MDG group. FJC displayed positive neurons of the MDG group, mostly in subgranular and granular layers, and showed a 4.1-fold elevation compared to the controls (*p* < 0.001; Figure 5). A similar picture was also detected when we analysed TUNEL+ cell positivity to estimate the number of cells with DNA damage in a whole cell population. We found a 3.5-fold increase in TUNEL+ cells in the subgranular and molecular layer in the MDG group (*p* < 0.01) in comparison to controls (Figure 5).

#### 3.2.2. Immunofluorescent Analysis

In the next set of experiments, we evaluated the impact of hHcy conditions on the DG by analysing the glial cellularity number. We used specific antibodies to detect microglia (Iba1) and GFAP to detect astrocytes.

**Figure 6:**
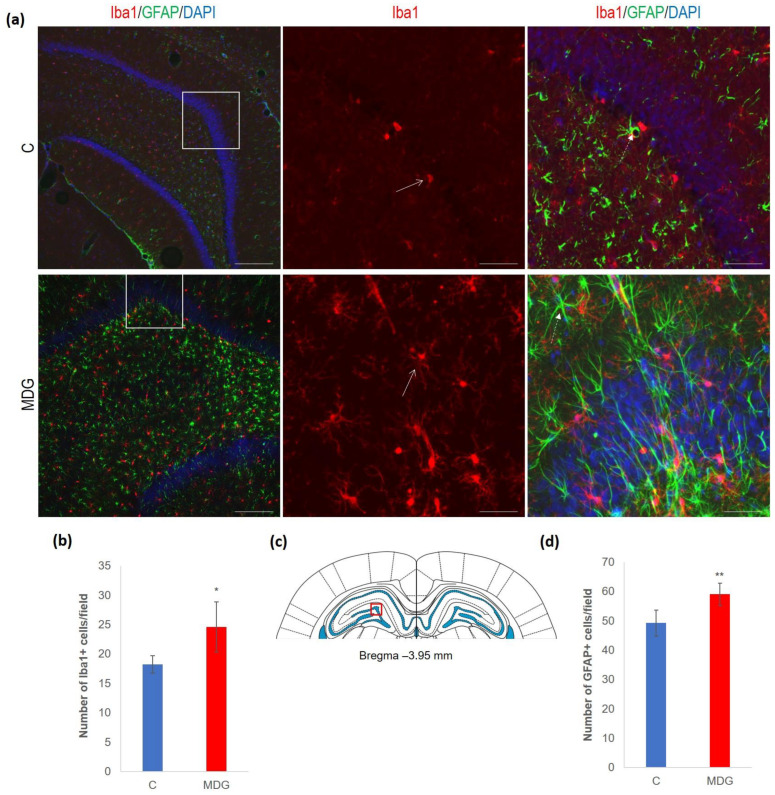
Illustrative micrographs of immunohistochemical detection of microglia (Iba1) and astrocytes (GFAP) in rat brain sections from the DG area of the hippocampus. (**a**) Fluorescent microphotographs of the hippocampus show the control and MDG groups at low magnification in the first row, and the second and third rows represent high magnification (rectangles) of the corresponding groups with specific antibody/overlay. Arrows indicate microglia, and dashed arrows point to astrocytes. Nuclei are counterstained with DAPI (blue). Scale bars = 200 and 50 μm; *n* = 8 per group. (**b**) The number of Iba1+ cells in the DG region of the hippocampus for the control and MDG groups. The Iba1+ significance of differences in group means was assessed using an unpaired *t*-test. The results are expressed as the mean ± SD for each group, with a sample size of *n* = 8, * *p* < 0.05 compared to the control group. The coronal rat brain section depicted in (**c**) has been redrawn from Paxinos and Watson [37], illustrating the DG photographed area (red rectangle). (**d**) The number of GFAP+ cells in the DG region of the hippocampus for the control and MDG groups. The GFAP+ significance of differences in group means was assessed using an unpaired *t*-test. The results are expressed as the mean ± SD for each group, with a sample size of *n* = 8, ** *p* < 0.01 compared to the control group.

We documented increased cellularity in the MDG group in comparison to the controls in both the microglia and astroglia cell types in all DG layers, likely as a tissue response to the neurotoxic Hcy conditions. An increase was more pronounced for microglia (35%, *p* < 0.05). The difference was less prominent for astrocytes and reached 19% (*p* < 0.01). An anticipated rise in glial cellularity in hHcy conditions was manifested by typical morphological patterns for both types of glial cells (Figure 6).

**Figure 7:**
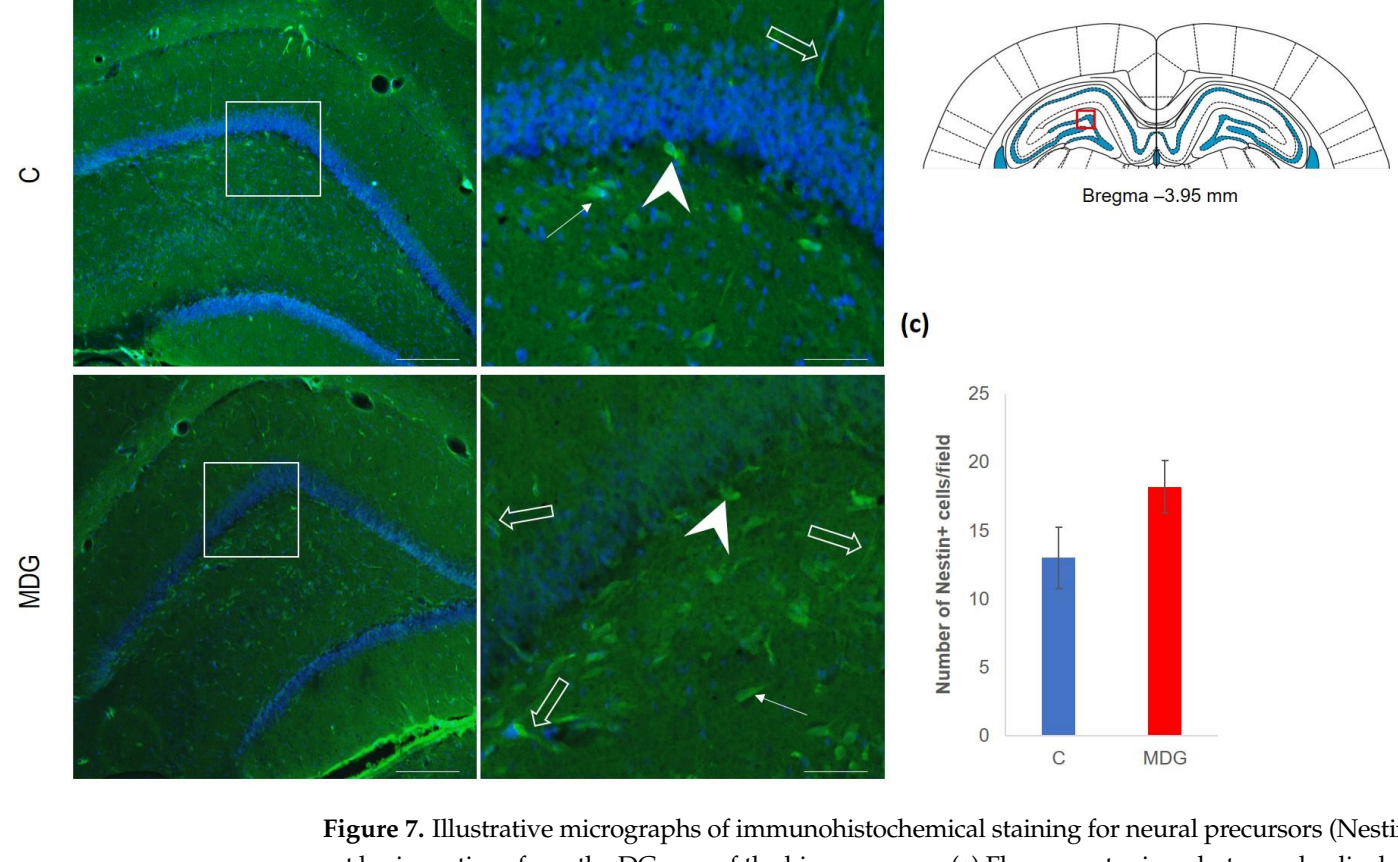
Illustrative micrographs of immunohistochemical staining for neural precursors (Nestin) in rat brain sections from the DG area of the hippocampus. (**a**) Fluorescent microphotographs displaying the control and MDG groups at low magnification in the first row, with a high magnification (rectangles) of the respective groups in the second row. Nestin-positive cells are indicated by arrows, while an arrowhead indicates the incorporation of Nestin+ cells into the granular layer of DG. Empty arrows point to blood capillaries. Nuclei are counterstained with DAPI (blue). Scale bars = 200 and 50 μm; *n* = 8 per group. The coronal rat brain section depicted in (**b**) has been redrawn from Paxinos and Watson [37], illustrating the DG photographed area (red rectangle). (**c**) The number of Nestin+ cells in the DG region of the hippocampus for the control and MDG groups. The Nestin+ significance of differences in group means was assessed using an unpaired *t*-test. The results are expressed as the mean ± SD for each group, with a sample size *n* = 8.

**Figure 8:**
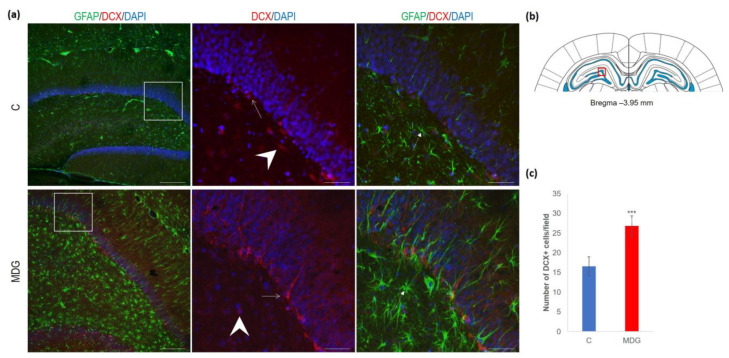
Illustrative micrographs of immunohistochemical staining for neuroblasts (DCX) with colocalisation of astrocytes (GFAP) in rat brain sections from the DG area of the hippocampus. (**a**) Fluorescent microphotographs show the control and MDG groups at low magnification in the first row, and the second and third rows display high magnification (rectangles) of the respective groups and specific antibody/overlay. Arrowheads indicate DCX positivity and dashed arrows point to astrocytes. Arrows depict the incorporation of neuroblasts into the granular layer of DG. Nuclei are counterstained with DAPI (blue). Scale bars = 200 and 50 μm; *n* = 8 per group. The coronal rat brain section depicted in (**b**) has been redrawn from Paxinos and Watson [37], illustrating the DG photographed area (red rectangle). (**c**) The number of DCX+ cells in the DG region of the hippocampus for the control and MDG groups. The DCX+ significance of differences in group means was assessed using an unpaired *t*-test. The results are expressed as the mean ± SD for each group, with a sample size *n* = 8, *** *p* < 0.001 compared to the control group.

The hippocampal DG region has an essential function in promoting neurogenesis in the adult rat brain. The question is whether enhanced neurodegenerative patterns as a response to hHcy can somehow interfere with this unique property of DG. The impact of hHcy on the differentiation of neural stem cells was analysed using different markers. Firstly, Nestin (a marker of neural stem cells), and, secondly, DCX (a marker of neuroblasts), both probably indicators of differentiation. Remarkably, in the MDG group, we documented an increase in Nestin+ positive neural stem cells. Nestin is known for its high affinity to the actively proliferating endothelium [38]. The hollow arrows in Figure 7 indicate the blood capillaries. Although there was an increase in the number of Nestin+ endothelial cells in the MDG group, the elevation was not statistically significant. As shown in Figure 7, Nestin+ positive cells are focally localised, but, in general, the elevation of the cellular number was not statistically significant. Remarkably, we observed a statistically significant increase in neuroblast formation in the MDG group, with the number of DCX+ cells rising by 48% (*p* < 0.001; Figure 8).

**Figure 9:**
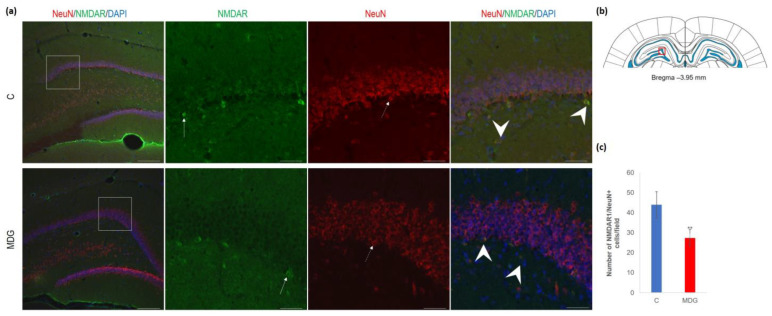
Illustrative micrographs of immunohistochemical detection of NMDA receptor subunit 1 (NMDAR1) with colocalisation of mature neurons (NeuN) in rat brain sections from the DG area of the hippocampus. (**a**) Fluorescent microphotographs of the hippocampus depict the control and MDG groups at low magnification in the first row, and the second to fourth rows indicate high magnification (rectangles) of the corresponding groups with specific antibody/overlay. Arrows indicate NMDAR1 positivity, dashed arrows point to mature neurons, and an arrowhead shows neurons with NMDAR1/NeuN positivity. Nuclei are counterstained with DAPI (blue). Scale bar = 200 and 50 μm; *n* = 8 per group. The coronal rat brain section depicted in (**b**) has been redrawn from Paxinos and Watson [37], illustrating the DG photographed area (red rectangle). In (**c**), the number of NMDAR1/NeuN cells in the DG region of the hippocampus is shown for both the control and MDG groups. The NMDAR1/NeuN+ significance of differences in group means was assessed using an unpaired *t*-test. The results are expressed as the mean ± SD for each group, with a sample size *n* = 8, ** *p* < 0.01 compared to the control group.

As shown by us [23,39] and others [14,18,29], hHcy impact on the selective brain region, such as the hippocampal CA1 region, is manifested by the neurodegenerative features resembling AD-like pathology. This study focused on the less vulnerable hippocampal region—DG. First, we used antibodies against NMDAR1 and NeuN for their common immune co-localisation in the mature DG neurons. We found a decreased number of NeuN+ cells and a lower number of NMDAR1 immunofluorescent cells, mainly in the granular layer of DG. The reduction in NMDAR1/NeuN+ cells was statistically significant and reached 37% (*p* < 0.01) in the MDG group (Figure 9).

**Figure 10:**
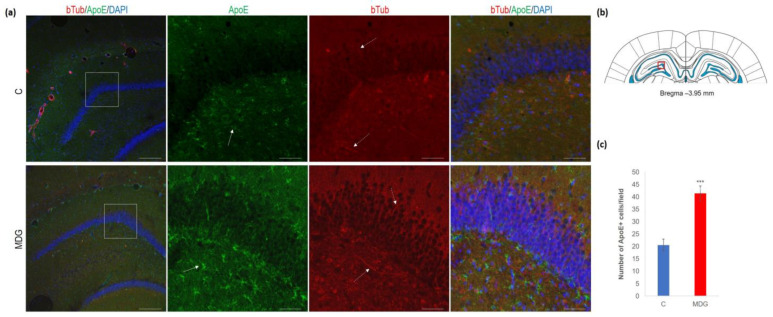
Illustrative micrographs of immunohistochemical detection of ApoE with colocalisation of bTub in rat brain sections from the DG area of the hippocampus. Fluorescent microphotographs (**a**) show the control and MDG groups with low magnification images in the first row and high magnification images (rectangles) in the second, third, and fourth rows of the respective groups and specific antibody/overlay. The arrows indicate ApoE-positive cells, and the dashed arrows point to bTub-positivity. Nuclei are counterstained with DAPI (blue). Scale bars = 200 and 50 μm; *n* = 8 per group. The coronal rat brain section depicted in (**b**) has been redrawn from Paxinos and Watson [37], illustrating the DG photographed area (red rectangle). In (**c**), the number of ApoE+ cells in the DG region of the hippocampus is presented for both the control and MDG groups. The ApoE+ significance of differences in group means was assessed using an unpaired *t*-test. The results are expressed as the mean ± SD for each group, with a sample size *n* = 8, *** *p* < 0.001 compared to the control group.

In the second series of experiments, we analysed ApoE immunosignal (as a putative AD risk factor) co-localised with bTub. We found an increase in ApoE+ cells (2-fold increase; *p* < 0.001), mainly in the subgranular molecular layers and the hilus of DG (Figure 10). Conversely, we observed an elevation in bTub positivity, indicative of cytoskeletal remodelling [22,40].

## 4. Discussion

An inadequate or excessive intake of Met in the diet is thought to be linked to various neurological and psychiatric disorders, along with age-related neurodegenerative conditions like AD and vascular dementia [6,19]. As a component of the hippocampus, the DG holds significant importance in associative memory. In the late stages of AD, it undergoes substantial changes due to the development of plaques, tangles, and neuronal loss. The neurotoxic effects of elevated Hcy, which acts as a potent NMDA receptor agonist, are particularly pronounced in the DG, where these changes may lead to disruptions in the cognitive and learning impairments observed in the early stages of AD [6,13,14,19,29,41].

Our model of mild hHcy is a widely used animal model for detecting the neurotoxic impact of increased levels of Hcy and is intended to partially mimic conditions of the general adult population where the mild hHcy prevalence is about 5–7% [29,42]. Our previous works showed remarkable neurodegenerative patterns in the highly vulnerable CA1 hippocampal region. This work focused on the less vulnerable dorsal hippocampal region—DG.

### 4.1. In Vivo Metabolic Changes in the Dorsal Hippocampus in Met-Diet-Induced hHcy Conditions

A non-invasive in vivo ^1^H MRS analysis was implemented to determine the impact of mild hHcy on the level of important brain metabolites in the dorsal hippocampus. The results of ^1^H MRS analysis displayed changes in the ratio of all chosen metabolites as a response to hHcy (Table 1).

Our experiments found a decline in tNAA/tCr, and tCho/tCr ratios with an elevation in mIns/tNAA, mIns/tCr, and tCho/tNAA in the dorsal hippocampus. Cr is recognised as a constituent present in all brain cells. At the same time, tNAA serves as a distinctive neuroaxonal marker, playing a vital role as an important organic osmolyte and a precursor in myelin/lipid synthesis [41]. The documented altered ratio reflects general pathologic severity, likely as a result of Hcy impact. This can be linked with one carbon unit metabolism disturbances and/or the consequent dysregulation of the myelinated tract, as tCho represents glial markers (mainly for oligodendrocytes). tCho levels are often increased during myelin degradation, and a rise in tCho compared to tNAA has been linked to cerebral infarctions, ongoing gliosis, and the processes of re-/de-myelinization [43]. Recent studies have demonstrated that tCho is associated with membrane turnover, directly influenced by Hcy removal [44]. In the study of Hooshmand et al. [41], an association of hHcy and the tCho/tNAA ratio with the development of dementia was found. Le Stunff and colleagues [45] demonstrated a connection between hHcy and ceramide metabolism in AD-like neurodegeneration. In our mild hHcy conditions, a decreased tCho/tNAA ratio can likely suggest disturbed cell membrane turnover, the myelination process, and/or neuroglial dyshomeostasis. This suggestion is also supported by an increased ratio of mIns/tNAA and mlns/tCr documented by us and others [46,47], and by our histomorphological analysis representing mIns as glial proliferation or a hyperplastic marker. Astrocytes that are pathologically activated and have larger cell volumes often show elevated levels of mIns [46]. This phenomenon was also observed in other brain pathologies such as status epilepticus [48], AD, and traumatic brain injury [47], and hHcy conditions can eventually be finalised to the development of AD-like features and behavioural disturbances. Another already described aspect of hHcy’s impact involves promoting neuronal tissue breakdown, oedema, and cell lysis, as well as potentially inducing a transition from oxidative energy metabolism to mitochondrial dysfunction [11,13,14,18,29]. Moreover, Hcy and its metabolites could impair one-carbon unit metabolism, protein N-homocysteinylation, epigenetic DNA, and histone dysregulation [3,4,49], causing a diversion from the finely tuned metabolic ratio balance.

### 4.2. Histomorphological Alterations in the DG in Met-Diet-Induced Mild hHcy Conditions

In our previous studies using the same hHcy model [22,23,24], we observed a significant increase in the number of neurons displaying neurodegenerative patterns, as indicated by CV (Nissl body staining) and TUNEL+ cells, specifically in the CA1 area of the rat hippocampus. Another feature of the high Met diet included increased hippocampal volume and the swelling of CA1 neurons [22,23,24]. Similar changes were also described in a recent paper by Tchantchou et al. [29], documenting high hippocampal vulnerability, increased oxidative stress, the suppression of tight junction protein expression, reduced neuronal cell density, and caspase 3 elevation in the rat hHcy model.

We describe here the alteration in number as well as in the morphology of both the astrocytes and the activated microglia, also in the less vulnerable hippocampal DG region. Richetin et al. [26] demonstrated that hillar astrocytes of the DG are sites with βD accumulated tau and altered mitochondrial dynamics and function. This led to a reduction in adult neurogenesis and impaired spatial memory performances. Interestingly, the brain’s proliferation and the activation of microglia, particularly around amyloid plaques, are prominent features of AD, playing dual protective or detrimental roles [40,50]. Microglial activation is an early event in AD pathology, even during the pre-plaque stage [50], observed in animal models [14,20,51] and human pathologies among individuals with mild cognitive impairment [2,9]. Recent research by Weekmann et al. [14] demonstrates that hHcy induction, as a neurotoxic risk factor, gives rise to the initial neuroinflammatory changes preceding amyloid alterations and cerebrovascular events associated with later neurodegenerative patterns.

Our research also revealed the presence of disrupted bTub as a response to the impact of hHcy. As it possesses a GTPase domain, bTub plays a significant role in microtubule dynamics, which might be directly or indirectly associated with the mechanisms involved in neurodegeneration, such as synaptic plasticity and the regulation of dendritic spine morphology [52], as well as memory formation [53]. Defective axonal transport is considered an early pathological event in AD, and experimental evidence suggests that the presence of amyloid-β peptide (Aβ) oligomers leads to the disruption of axonal transport in cultured neurons [51].

Taking together all the above facts, we supposed that mild hHcy conditions also lead to microtubule disorganisation, and this finding correlates with results from the MRS analysis.

### 4.3. Neurogenesis in Met-Diet-Induced hHcy Conditions

Impaired or disturbed hippocampal neurogenesis is part of the etiopathogenesis of neurodegenerative diseases, including AD. However, data are somewhat controversial in documenting increased or suppressed cell proliferation and show the vital roles of neuroinflammation and microglia activation [54,55,56].

In the literature, only a few data are available to describe the impact of hHcy on hippocampal neurogenic activities. Owens et al. [55] found that the relative level of methylenetetrahydrofolate reductase does not significantly impact neurogenesis in three-week-old mice in response to an increased level of Hcy. Our results, which document activated cellular proliferation in hHcy conditions, can partially resemble the outcomes of Sakurai et al. [56], detecting an increase of DCX-positive neuroblasts in the piriform cortex of rats with induced status epilepticus. Currently, we have no direct explanation for our results; one can speculate about the possible compensatory mechanism activated by the loss of neurons in the neurotoxic hHcy conditions [54]. Moreover, the presence of a higher number of Nestin+ endothelial cells suggests an increased turnover of the capillary lining as a typical cerebrovascular consequence of hHcy [14].

### 4.4. Alterations in NMDAR and ApoE in the DG in Met-Diet-Induced Mild hHcy Conditions

ApoE lipoproteins and the *ApoE4* genotype are, with metabolic and environmental factors such as Hcy, tightly linked to the risk of vascular atherogenic changes, which can lead to dementia and AD. ApoE-containing lipoproteins are endocytosed into cells via ApoE receptors, which, interestingly, closely communicate with the NMDA receptors [57].

The impact of hHcy on grey matter volume was documented as partially dependent on vitamin B12 (cofactor catalysing the conversion of Hcy to Met). It may also be influenced by the ApoE genotype [58]. In the in vitro study of Trusca et al. [59], it was shown that extremely elevated levels of Hcy (250–750 μmol/L) led to a 2–3-fold reduction in ApoE mRNA in cultured cells. In comparison, lower concentrations of Hcy (100 μmol/L) primarily affected ApoE gene expression through decreased *ApoE* promoter activity, although not significantly. In addition, the ApoE4 genotype is linked with pronounced microglia-mediated neuronal damage, tau pathology [60], and the impaired clearing of Aβ42 [61].

As documented by previous studies, Hcy (or its oxidation forms) also affects the functional abilities of NMDARs localised in different brain cells with the outcome of increasing calcium influx, activating a pro-inflammatory cascade, and accumulating reactive oxygen species [62]. As a result, it can be followed by an increase in nuclear factor kappa B, regarded as one of “the master regulators of the expression of inflammatory genes” [49,63]. Furthermore, the Hcy binds to the NMDA receptor, and this interaction primarily depends on the presence of glycine (again, part of one-carbon metabolism). A higher glycine level occurs in many brain pathologies, such as ischemia or trauma, and a noteworthy increase in astrocytes (primary glycine source in the hippocampus; [64]) was also documented by our MRS and immunofluorescence results. Conditions with increased glycine promote the agonistic function of Hcy to the NMDA receptor even at a low Hcy concentration (i.e., Hcy = 10 µmol/L), exerting elevated excitatory action and enhancing calcium influx [63]. Additionally, previous studies have demonstrated that chronic neuroinflammation leads to the loss of NMDARs [65], and a decrease in NMDAR1 has been observed after the death of CA1 pyramidal cells in the hippocampus [66]. Moreover, any effect of NMDA receptors is followed by remarkable synaptic alteration [67]. We document in this study that the hHcy induces a decrease in the NMDAR1 expression localised in the granular layer of DG. Interestingly, in another study on a similar hHcy model, this condition was tightly associated with memory decline [29]. Taken together, our results show that mild hHcy conditions are linked with ApoE upregulation and alterations in the NMDA receptors, which points to proposed disturbances in synaptic functions and/or in mutual signalling processes [57,68].

**Figure 11:**
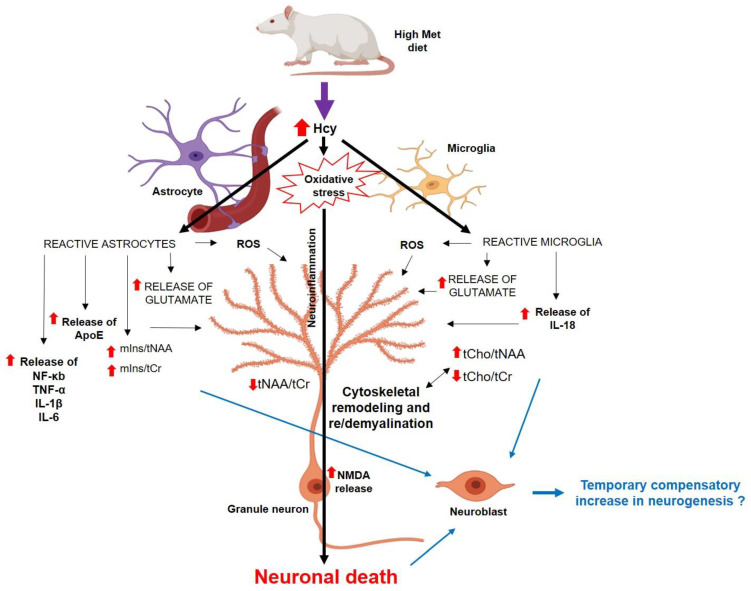
The scheme of possible mechanisms of how the hHcy affects the DG cells [22,23,54,55,56,69,70], partially created with https://app.biorender.com/ (accessed on 25 June 2023). Abbreviations: **↑**—increased concentration/ratio; ↓—decreased concentration/ratio; IL—interleukin; NF-κb—nuclear factor-kappa beta; ROS—reactive oxygen species; TNF-α—tumour necrosis factor.

In summary, we have shown here that mild hHcy impact is linked with the metabolic, histomorphological, as well as ApoE and NMDAR1 alterations in the less vulnerable dorsal hippocampus—the DG region. These conditions are also manifested by reduced neuronal density in DG (Figure 11), which altogether could result in an accelerated decline in working memory performance, as shown by previous studies on similar animal models [13,18,29,41]. Hcy itself, as a part of one carbon unit metabolism, or its oxidative metabolites may additionally impair processes such as DNA repair and methylation, as well as reduce the availability of neurotransmitters and the metabolism of phospholipids and myelin [49]. This may underly histological features of AD-like pathology and presumably could lead to later cognitive dysfunction [58,68]. Our findings suggest that hHcy is a candidate with critical epigenetic and neurotoxic properties that tremendously modulate pathological heterogeneity in the less vulnerable hippocampal DG also. Therefore, the hHcy condition in human pathology deserves attention as a putative therapeutic target.

## 5. Conclusions

In conclusion, Hcy remains a biological marker with limitations and no definitive resolution for most neuropathologies [63]. Meta-analyses have shown a causal relationship between plasma total Hcy and the risk factor for AD [1,2,5]. More than 40% of AD patients have elevated plasma Hcy levels, associated with faster neural atrophy than those with normal Hcy levels [71]. Additionally, elevated Hcy levels can forecast cognitive decline in healthy elderly individuals [9,72,73,74]. Therefore, Hcy also exhibits promise as a predictive indicator for AD, and counteracting Hcy-induced neurotoxicity might emerge as an innovative approach for preventing and treating AD [2,6,73,74,75].

## Figures and Tables

**Table 1:** The ^1^H MRS performed in the dorsal hippocampus of both MDG subgroups (before and after Met diet). The relative concentrations (mean ± SD) of ^1^H MRS metabolite ratios (tNAA/tCr, mIns/tNAA, mIns/tCr, tCho/tNAA, tCho/tCr) were evaluated for the prior_MET and after_MET animal groups. Paired 2-tailed *t*-tests (SPSS software, version 15.0; Chicago, IL, USA) were used to obtain *p*-values, indicating the statistical differences in metabolite ratios between the subgroups.

Dorsal Hippocampus				
	Animal Group	prior_Met /*n* = 8/ (Mean ± SD)	after_MET /*n* = 8/ (Mean ± SD)	prior_MET vs. after_MET *p*-Value
MRS Ratio				
tNAA/tCr		0.958 ± 0.144	0.888 ± 0.074	0.107
mIns/tNAA		0.442 ± 0.176	0.493 ± 0.140	0.584
mIns/tCr		0.408 ± 0.129	0.438 ± 0.135	0.710
tCho/tNAA		0.216 ± 0.053	0.223 ± 0.056	0.735
tCho/tCr		0.203 ± 0.037	0.195 ± 0.040	0.632

## Data Availability

Data sharing not applicable. No new data were created or analysed in this study. Data sharing is not applicable to this article.

## Abbreviations

^1^HMRS	Proton MRS
ApoE	Apolipoprotein E
CV	Cresyl violet
DCX	Doublecortin
DG	Dentate gyrus
FJC	Fluoro-Jade C
Hcy	Homocysteine
hHcy	Hyperhomocysteinaemia
MDG	Met diet group
Met	Methionine
MR	Magnetic resonance
MRS	MR spectroscopy
NeuN	Neural nuclei
NMDAR1	N-methyl-D-aspartate receptor 1
TUNEL	Terminal deoxynucleotidyl transferase dUTP nick end labelling
